# Low-dose radiation, scientific scrutiny, and requirements for demonstrating effects

**DOI:** 10.1186/1741-7007-11-92

**Published:** 2013-08-30

**Authors:** Anders Pape Møller, Timothy Alexander Mousseau

**Affiliations:** 1 Laboratoire d’Ecologie, Systématique et Evolution, CNRS UMR 8079, Université Paris-Sud, Bâtiment 362, F-91405, Orsay Cedex, France; 2 Department of Biological Sciences, University of South Carolina, Columbia, SC 29208, USA

## Abstract

Recent nuclear accidents have prompted renewed interest in the fitness consequences of low-dose radiation. Hiyama *et al*. provided information on such effects in the Japanese pale grass blue butterfly in a paper that has been viewed more than 300,000 times, prompting a barrage of criticism. These exchanges highlight the role of scrutiny in studies with potential effects on humans, but also raise questions about minimum requirements for demonstrating biological effects.

## 

Natural background radiation varies by more than a factor of 300 with significant negative effects on mutation, immunology and disease [1]. Low-dose radiation has been known to have negative consequences for living beings for almost 100 years [2]. Indeed, background radiation causes the death of tens of thousands of humans annually [3]. These ‘natural’ effects may be exacerbated by the 23 nuclear accidents recorded during the last century, ranging from 4 to 7 on the International Nuclear Event Scale [4]. A level 4 accident is defined as having local consequences involving a release of radioactive materials exceeding 0.1% of core inventory with a high probability of significant public exposure and at least one death from radiation. Only Chernobyl and Fukushima have reached the highest level of 7 for a core meltdown with a major release of radioactive materials and predicted widespread health and environmental impacts, although another major nuclear accident is predicted to occur during the next 50 years [4]. Hence it is not surprising that there is a renewed proactive interest in the consequences of low-dose radiation on all organisms, including humans, under field conditions.

Surprisingly, the health consequences of nuclear accidents are poorly understood [5]. For example, the range of estimated excess human deaths due to the Chernobyl accident varies from less than 50 [6] to several hundred thousand [7], with scientists associated with the nuclear industry and governmental oversight bodies consistently reporting low estimates and ‘independent’ scientists generally providing larger estimates. Recently, Hiyama *et al*. [8] presented the most extensive information so far on abnormalities and survivorship caused by chronic low-dose radiation in any species, in this case the pale grass blue butterfly *Zizeeria maha* under field conditions in Japan.

## Levels of scientific scrutiny

The paper by Hiyama *et al*. [8] has been viewed a staggering 302,400 times as of 24 July 2013. The main findings across ten localities that included Fukushima were elevated frequencies of morphological abnormalities in butterflies from Fukushima, a higher frequency of abnormalities in the second and the third generation compared to the first, which was not exposed to radiation, and an increase in abnormalities for butterflies treated with internal and external radiation exposure under laboratory conditions (Table 1). Interestingly, elevated frequencies of abnormalities were associated with smaller size and depressed survival rates as expected for deleterious mutation. Minor morphological abnormalities are well known to increase in frequency under environmental stress, including exposure to radiation, as is the link between abnormalities and elevated risk of death [9].

**Table 1:** **Predictions and statistical tests for effects of low-dose radiation on pale grass blue butterflies (Hiyama ***et al. *[8]**)**

**Analyses**	**Expectations**	**Findings**
Spatial variation	If radiation was the causative agent, we should expect more abnormalities in samples from Fukushima	Increased frequency of abnormalities at Fukushima
Temporal variation	If radiation was the causative agent, we should expect fewer abnormalities in samples from the first generation not exposed to radiation compared to second and third generations	Increased frequency of abnormalities in second and third generations
Radiation experiment	If radiation was the causative agent, we should expect more abnormalities in animals exposed to internal and external radiation in the lab	Increased frequency of abnormalities following lab exposure to radiation, but not in controls
Survival rate	If radiation had negative effects, we should expect delayed growth and reduced survival	Reduced growth and survival in irradiated samples

The approaches adopted by Hiyama *et al*., the sample sizes, and the analyses are standard for ecological and evolutionary studies, so there is no major reason for objections. Still, numerous objections were raised to these interesting findings. In a rebuttal Hiyama *et al*. [10] provided extensive evidence that supports their initial conclusions (Table 2). In particular, they have shown five important findings: the color patterns obtained in Fukushima differed from color patterns produced by aberrant temperatures and sibling crosses; the minor morphological abnormalities were not present at high frequency in Fukushima before the accident, as shown by older specimens in collections; the abnormal traits were heritable; mutation accumulation occurred from May to September 2011; and finally, positive controls produced normal adults. These findings and approaches are well within expectations for rigorous scientific research.

**Table 2:** **Predictions and statistical tests for effects of low-dose radiation on pale grass blue butterflies (Hiyama ***et al. *[10]**)**

**Analyses**	**Expectations**	**Findings**
Color patterns specific for radiation	If radiation is a unique environmental stressor, we should expect specific effects of radiation	Color patterns were specific for radiation, and differed from those caused by temperature and crosses between siblings
Temporal variation predating the accident	If radiation is the causative agent for abnormalities, there should be fewer abnormalities from Fukushima before the accident	Lower frequency of abnormalities at Fukushima before than after the accident
Heritability of minor morphological abnormalities	If the abnormalities are caused by germline mutations, we should expect these to be transferred to the next generation	Offspring resembled their parents with respect to abnormalities in random crosses
Mutation accumulation	If mutations accumulate over time, there should be an increase in the frequency of abnormalities from first over second to third generation	Increase in frequency of abnormalities across generations
Positive controls	Controls reared in the laboratory, but not exposed to radiation should resemble animals from control areas with respect to abnormalities	Similar frequency of abnormalities in positive controls and animals from uncontaminated areas

But the great strength of this study rests with the use of lab-based experimental manipulations of radiation that capture the genetic and phenotypic consequences observed in the wild-caught populations. In the absence of such experimental manipulations this study would be far less convincing given the small sample sizes of field collections, and lack of replication of populations within contaminated areas with the result that a dose–response relationship could not be characterized for wild populations. However, the use of experimental manipulations provides unambiguous support for the hypothesis that radioactive fallout is the likely cause of the abnormalities observed in the field and this effectively counters all of the major criticisms that have been levied at this study.

## Minimum requirements for demonstrating low-dose effects

The papers by Hiyama *et al*. [8,10] raise questions about the minimum levels of scientific scrutiny for demonstrating effects of low-dose radiation, but also other environmental health problems. We consider the approach taken by Hiyama *et al*. [8,10] to be fully adequate for an initial investigation of association between radiation, mutations, and their phenotypic effects. However, the following additional research questions should be addressed (Table 3). First, numerous ecological phenomena are density-dependent, and that is also the case for minor morphological abnormalities [9]. Given the increased levels of mortality in contaminated areas, we consider the frequency of abnormalities in contaminated areas are likely to be under-estimates. Larger scale sampling of populations would likely address this issue. Second, because animals are smaller and have more abnormalities in contaminated areas, they might be more likely to be caught. Characterization of capture efficiency could help to address this question. Third, whole-genome sequencing of butterflies from specimens irradiated in the laboratory and collected from control areas and the first, second and third generations in Fukushima would unequivocally demonstrate the association between irradiation and mutation accumulation, although we recognize that achieving such a goal would necessarily require a large financial investment, which has not generally been available for such studies in the past.

**Table 3:** Suggestions for future research

**Analyses**	**Expectations**
Density-dependence	Greater frequency of abnormalities at high population density
Capture probability	Minor abnormalities and smaller size should increase the probability of capture and hence the frequency estimate
Genome-wide sequencing to quantify mutations	Greater frequency of mutations in specimens from Fukushima than in control areas, increasing frequency across generations due to mutation accumulation, and greater frequency in irradiated animals from the laboratory compared to controls.

Finally, Hiyama *et al*. [10] emphasize that their study deals with the consequences of chronic rather than acute exposure to radiation, as will all studies of this phenomenon under field conditions. Again, this raises the question of why scientists working for the nuclear industry and in national and international laboratories have not already conducted extensive research on the consequences of such chronic radiation exposure. Unfortunately, funding for fundamental research in these areas in Japan, Europe and the US has never been large and has recently been scaled back [11]. Hopefully, the wide interest in such questions provoked by Hiyama *et al*. [8] will provide justification for greater investment in this research area of increasing societal relevance.
